# Real-time citrus variety detection in orchards based on complex scenarios of improved YOLOv7

**DOI:** 10.3389/fpls.2024.1381694

**Published:** 2024-07-01

**Authors:** Fuqin Deng, Jianle Chen, Lanhui Fu, Jiaming Zhong, Weilai Qiaoi, Jialong Luo, Junwei Li, Nannan Li

**Affiliations:** ^1^ School of Electronic and Information Engineering, The Wuyi University, Jiangmen, China; ^2^ School of Computer Science and Engineering, Faculty of Innovation Engineering, Macau University of Science and Technology, Macau, China

**Keywords:** object detection, YOLOv7, citrus variety detection, XinHui citrus, GSConv, BiFormer

## Abstract

Variety detection provides technical support for selecting XinHui citrus for use in the production of XinHui dried tangerine peel. Simultaneously, the mutual occlusion between tree leaves and fruits is one of the challenges in object detection. In order to improve screening efficiency, this paper introduces a YOLO(You Only Look Once)v7-BiGS(BiFormer&GSConv) citrus variety detection method capable of identifying different citrus varieties efficiently. In the YOLOv7-BiGS network model, initially, the BiFormer attention mechanism in the backbone of the YOLOv7-based network strengthens the model’s ability to extract citrus’ features. In addition, the introduction of the lightweight GSConv convolution in place of the original convolution within the ELAN of the head component effectively streamlines model complexity while maintaining performance integrity. To environment challenge validate the effectiveness of the method, the proposed YOLOv7-BiGS was compared with YOLOv5, YOLOv7, and YOLOv8. In the comparison of YOLOv7-BiGS with YOLOv5, YOLOv7, and YOLOv8, the experimental results show that the precision, mAP and recell of YOLOv7-BiGS are 91%, 93.7% and 87.3% respectively. Notably, compared to baseline methods, the proposed approach exhibited significant enhancements in precision, mAP, and recall by 5.8%, 4.8%, and 5.2%, respectively. To evaluate the efficacy of the YOLOv7-BiGS in addressing challenges posed by complex environmental conditions, we collected occluded images of Xinhui citrus fruits from the Xinhui orchard base for model detection. This research aims to fulfill performance criteria for citrus variety identification, offering vital technical backing for variety detection endeavors.

## 1 Introduction

Agriculture is the primary means of subsistence and revenue generation (Naga et al., 2024). In 2022, the output value of XinHui tangerine peels industry exceeds 19 billion yuan, which is 31% higher than that of 2021. XinHui citrus grown in XinHui area is the only raw material for making XinHui tangerine peels (Pan, 2023). A variety of citrus including XinHui citrus, Emperor citrus and so on are grown in large citrus orchards. Low-quality or counterfeit tangerine peel may be produced when other citrus varieties are used. Not only are consumer interests compromised, but the reputation of the growers is also damaged as a result. Different citrus varieties possess entirely different economic values. Hence, during citrus harvesting, a large number of experienced fruit pickers are required to be employed by variety. This practice not only diminishes harvesting efficiency (Wang et al., 2023) but also escalates labor costs (Tang et al., 2024), ultimately reducing the growers’ profits. However, the fertilizer requirements vary significantly among different citrus varieties. Therefore, to ensure both yield and quality, a scientific fertilization process tailored to the citrus variety is essential during automated fertilization. Therefore, the trend towards intelligent orchard management is gaining momentum (Wu et al., 2023).

In agricultural production, the adoption of robot-assisted harvesting (Ye et al., 2023) and fruit variety classification is key for achieving intelligent orchard management (Koirala et al., 2019). Agricultural robots excel at performing highly repetitive tasks, which makes them well-suited for the monotonous and lengthy labor involved in orchard management (Fu et al., 2022). The foundation for agricultural robots to achieve variety identification and automated harvesting is formed by visual detection systems, serving as the core of intelligent orchard management (Chen et al., 2024). However, fruit detection faces some challenges. Firstly, misjudgments can easily occur due to the subtle visual differences between different citrus varieties. Secondly, randomly distributed growth of citrus fruits results in overlapping fruits and obstruction by tree branches and leaves. Therefore, to identify the fruit types, researchers have conducted numerous explorations. In recent years, with the development of deep learning technology, detection methods based on deep learning have been gradually applied in the agricultural domain. Among them, these methods are primarily divided into two-stage algorithms and single-stage algorithms. Two-stage algorithms involve neural network models that are relatively large, such as Fast R-CNN (Girshick, 2015), R-FCN (Dai et al., 2016), Mask r-cnn (He et al., 2017), SPP-Net (Purkait et al., 2017), FEANet (Deng et al., 2021). These algorithms have slower computational speeds and cannot meet the real-time detection requirements. Single-stage algorithms mainly include YOLO (You Only Look Once) (Redmon et al., 2016) and Single Shot Multibox Detector (SSD) (Liu et al., 2016). Chen et al. (2022) proposed a method that combines YOLOv5 with visual saliency maps, which uses a visual saliency detection algorithm to identify the ripeness category of citrus by YOLOv5 detection. Continuously refining deep learning methods allows for better detection of obscured objects. Hou et al. (2022) utilized the YOLOv5s method improved by binocular vision to detect and locate mature citrus fruits under uniform lighting, achieving a recall rate of 98%. However, this method did not perform variety detection on the target fruit. Sozzi et al. (2022) validated the YOLOv4 algorithm as the optimal method for balancing speed and accuracy by detecting red and white grapes. Riaz et al. (2020) used neural networks to classify four varieties of oranges, with an accuracy rate of only 80%. Rodríguez et al. (2018) used convolutional neural networks to classify plum varieties with an accuracy of 91%, but did not consider the complex environment in orchards. But the detection accuracy is not high enough. Zhao et al. (2023) introduced the IMVTS model for classifying tea buds of different varieties with an accuracy of 99.87%. Despite notable advancements in object detection through deep learning, challenges persist in citrus variety classification attributed to the intricate orchard backgrounds and subtle variations in appearance size among different citrus varieties. Therefore, to enhance the model’s feature extraction capability, this paper chose the YOLOv7 (Wang et al., 2023) network as the base model and constructed the YOLOv7-BiGS network. In this network, the BiFormer attention mechanism (Zhu et al., 2023) has been incorporated to enhance the ability to extract citrus texture features, aiming to improve the capability of the backbone network in citrus feature extraction. To reduce the complexity of the network model while maintaining accuracy, the GSConv module (Li et al., 2022) has been integrated into the YOLOv7 network. The detection results of YOLOv7-BiGS are analyzed using multiple metrics, and the performance of YOLOv7-BiGS is experimentally compared with the performance of other major object detection models. The effectiveness of the GSConv module and BiFormer attention mechanism was verified through ablation experiments.

The main contributions of this study are as follows: (1) Model Innovation: Our innovation lies in the development of the YOLOv7 BiGS model, specifically designed to excel in citrus variety detection amidst complex backgrounds. Through meticulous optimization of the network architecture and the integration of an advanced attention mechanism, our model achieves remarkable accuracy even in challenging scenarios. (2) Datasets Development: We constructed a comprehensive dataset comprising real-world citrus fruit images captured in orchard environments. This dataset will serve as a valuable resource for training and evaluating the model, providing diverse and realistic data for optimal performance assessment. (3) Performance Enhancement: Leveraging the combined power of GSConv and the BiFormer attention mechanism, we have successfully elevated the detection accuracy and computational efficiency of our model. This strategic integration enhances the model’s capability to accurately identify citrus varieties while optimizing resource utilization. (4) Real-Time Application Potential: Our method boasts a compact model size and superior computational efficiency, positioning it as a viable solution for real-time citrus fruit detection applications. With its streamlined architecture and rapid processing capabilities, our model holds significant promise for seamless integration into practical deployment scenarios. This technological support contributes significantly to intelligent management in citrus orchards and ensures the supply of raw materials for citrus peel production.

## 2 Image data pre-processing

### 2.1 Image data acquisition

**Figure 1:**
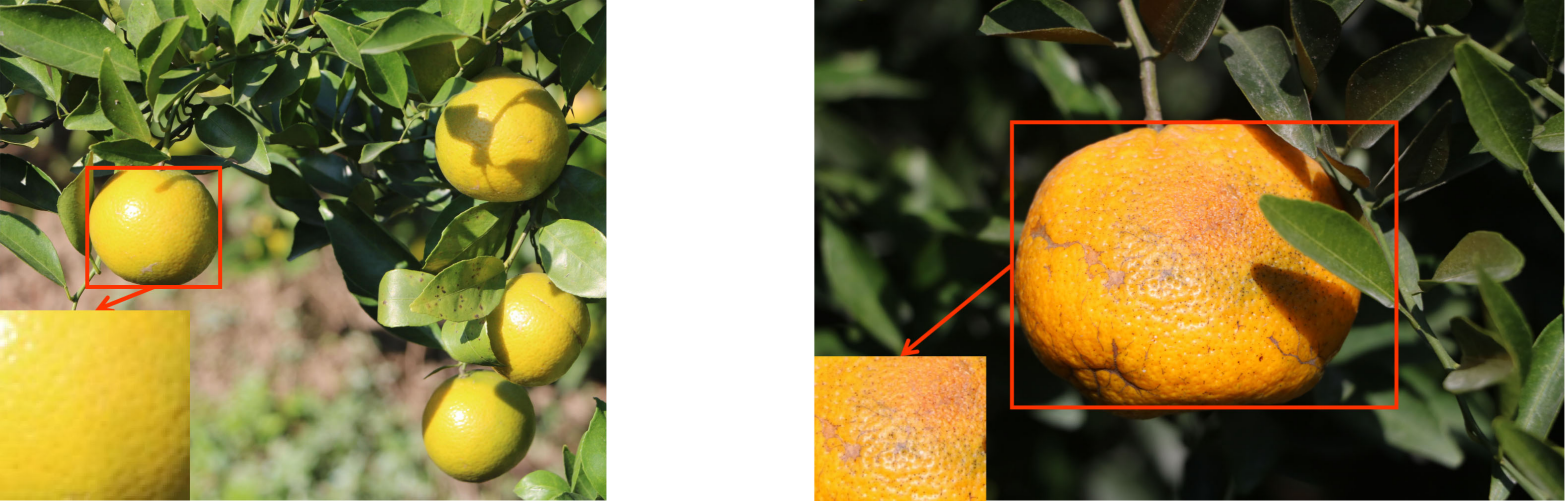
Comparison of citrus texture collected from orchards. **(A)** Xinhui citrus. **(B)** Emperor citrus.

In this study, images were captured using Canon EOS 760D cameras from 1 pm to 4 pm on sunny days at the MeiQie Orchard in XinHui District, Jiangmen City, Guangdong Province, China, for two citrus species: XinHui citrus and Emperor citrus. As shown in **Figure 1**, **Figure 1A** shows XinHui citrus, and **Figure 1B** shows Emperor citrus. From the appearance, it can be seen that the texture features of Emperor’s citrus are relatively delicate, while the texture features of Xinhui citrus are relatively rough. A total of 400 images of XinHui citrus and Emperor citrus were obtained, with uneven conditions such as leaf occlusion, overlap occlusion, branch occlusion, similar visual appearance to the background image, dense targets, branch occlusion, back light, front light, side light, and other fruit natural scenes, and saved in JPG format.

### 2.2 Data labeling

The annotated dataset is manually annotated with citrus fruits using LabelImg and saved in YOLO format. The annotated rectangular frame conforms to the outline of the fruit. When drawing a rectangular box, if the object is obstructed by branches, leaves, or citrus fruits, the contour is drawn based on experience to depict the actual size of the object. When labeling citrus fruits, only the citrus fruits with clear textures in the images are labeled, and situations such as blurred textures, severe occlusion, and dim backlight are not labeled, totaling 400 images.

### 2.3 Data augmentation

**Figure 2:**
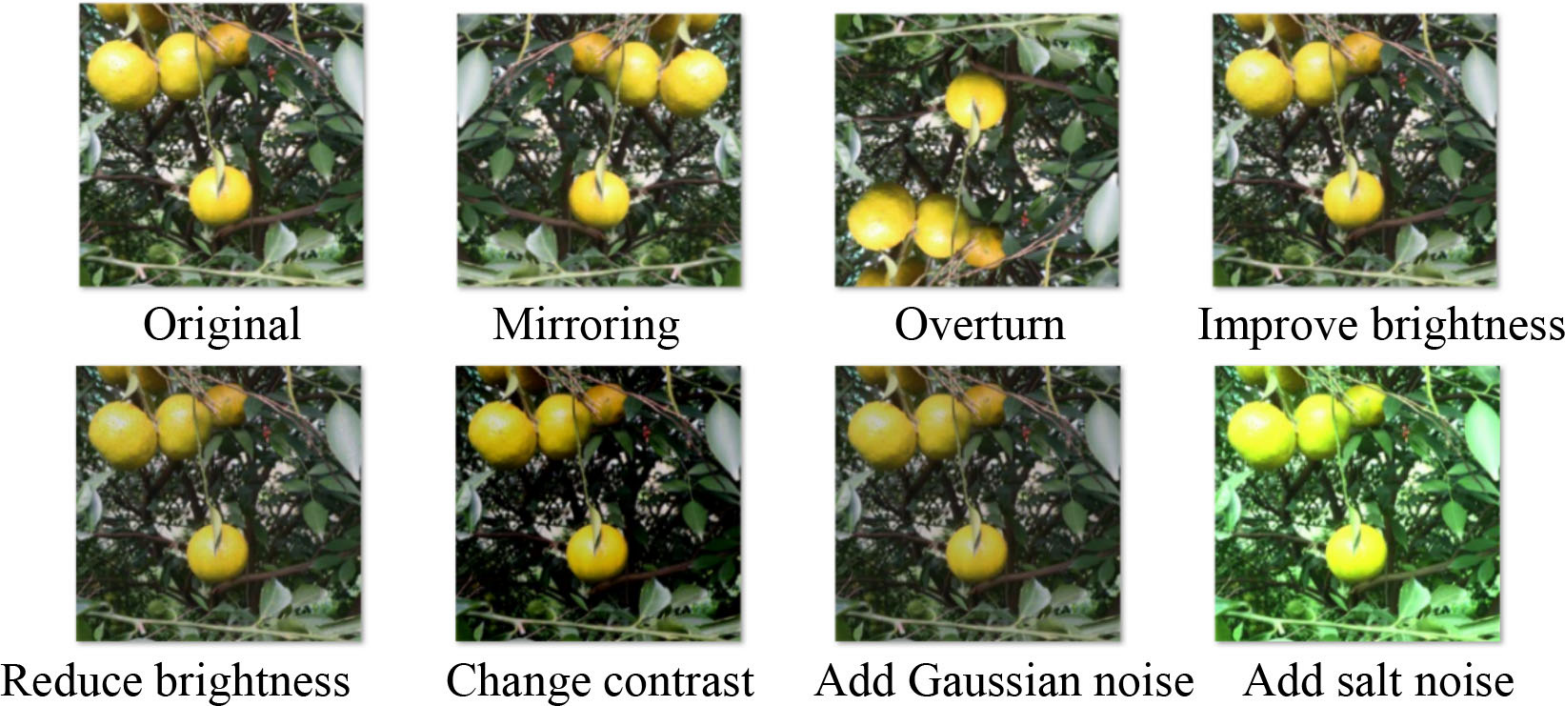
Citrus image after data augmentation.

Data augmentation is a commonly used technique that increases the diversity and richness of training data through a series of transformations and expansions, helping to enhance the model’s generalization ability. The data augmentation methods include image rotation, flipping, cropping, scaling, changing image brightness, changing contrast, adding Gaussian noise, adding salt noise, and other operations. The images with augmented data for the collected images and annotations are depicted in **Figure 2**.

The amount of data in the training set has increased from 400 to 3060, with a total of 297 sheets in the test set and 19 sheets in the validation set, respectively. The operation of data augmentation can simulate various changes and noise in actual scenarios, thereby making the model better adapt to different situations.

## 3 Methods

### 3.1 YOLOv7-BiGS

The YOLO algorithm’s classification ability and relatively high accuracy have great advantage in object detection networks, so the YOLO series has been widely applied in the field of agriculture. YOLOv7 can be trained and can be used to detect when the image quality is unsatisfactory due to image blurring caused by shooting, foliage occlusion, and fruit overlap (Yuan, 2023). Compared with the two-stage algorithms, YOLOv7 is able to achieve high accuracy and high efficiency, and possesses strong comprehensive performance for fast and accurate object identification and variety detection. Therefore, in order to achieve the high accuracy in XinHui citrus variety detection, YOLOv7 is optimized and improved in this paper, which is capable of recognizing citrus with incompletely exposed fruits due to leaf occlusion or fruit overlap. The size of input images in the YOLO model is 640 × 640. The YOLO model uses 3 × 3 or 1 × 1 convolution kernels. These convolution kernel sizes are selected based on empirical evidence and computational considerations. The 3 × 3 convolution kernel captures spatial information of local regions in the input image, while the 1 × 1 convolution kernel performs channel level operations to adjust the depth of feature mapping. In the experiments, it is shown that the YOLOv7-BiGS model achieved better results in citrus variety detection.

**Figure 3:**
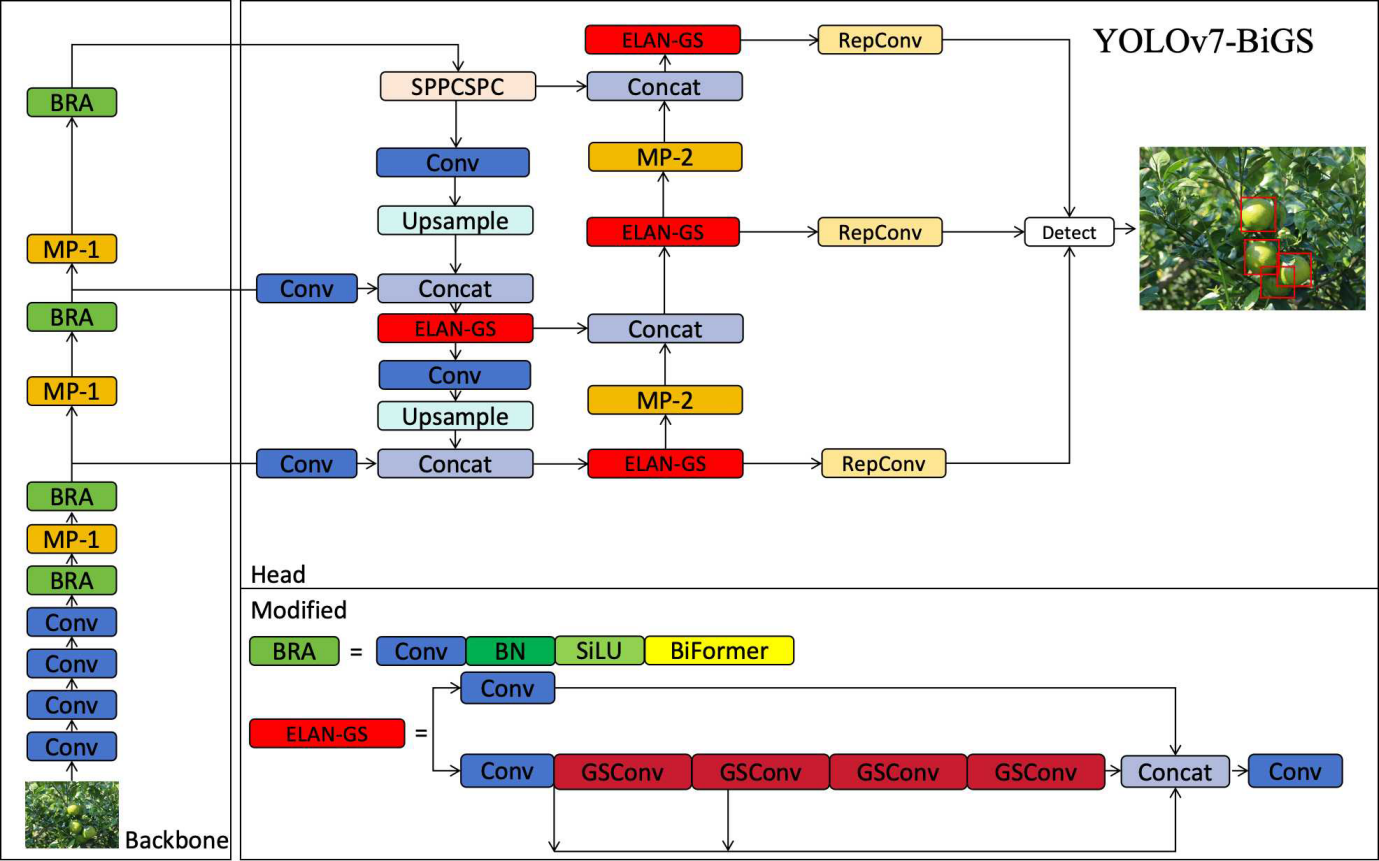
YOLOv7-BiGS network structure.

The network structure of YOLOv7-BiGS primarily consists of two parts: Backbone and Head. The function of extracting image features is mainly implemented within the Backbone. The improved BRA module replaces the ELAN module in the Backbone. In the BRA module, a BiFormer attention module is added to enhance the neural network model’s feature extraction capability through an attention mechanism. Considering the characteristics of feature fusion, there still exist a significant number of model parameters during the feature fusion process, impacting the speed of fusion. The ELAN module in the Head is replaced by ELAN-GS. Within ELAN-GS, the standard Conv module of ELAN is substituted with the GSConv module, reducing the computational load of the model. The enhanced network structure of YOLOv7-BiGS is illustrated in **Figure 3**.

The Backbone network is the feature extraction part of the YOLOv7-BiGS model and this part extracts high-level features from the original image. The Backbone network here consists of a series of convolutional layers, pooling layers, and a BiFormer layer. These layers are stacked in sequence. The Conv module consists of convolution, batch normalization, and SiLU activation functions to extract features. The Backbone network starts with a convolutional layer with a 3x3 convolutional kernel and a stride of 1, which is used to process the input image. On the next layers, the Backbone network gradually increases the characteristic depth through the convolutional layers. These convolutional layers include layers with different numbers of filters and various sizes of convolutional kernels to gradually extract more complex features. After some of the convolutional layers, the backbone network includes a Max Pooling layer to reduce the size of the feature map and enable the network to capture information at different scales. The BiFormer layer integrates BiFormer attention mechanisms, convolution operations, and routing operations to sharpen the model’s attention on specific features and amalgamate information from diverse feature layers. As the backbone network progresses, multiple feature layers undergo concatenation, fostering the accumulation of comprehensive multi-scale information. This amalgamation is pivotal in extracting intricate feature representations essential for subsequent processing by the head network, ultimately culminating in precise object detection results.

The Head network is the output generation part of the YOLOv7-BiGS model, transforming the feature mappings extracted from the backbone network into the output for object detection. The Head network consists of a series of convolutional layers, an upsampling layer, a concatenation and a customized ELAN-GS layer, as well as an object detection layer. The Head network consists of multiple convolutional layers with different numbers of filters and various convolutional kernel sizes, and these layers are used to process the feature mapping from the neck network. The Head network zooms in on the feature maps on the upsampling layer to merge them with different scales of feature maps from the neck network. The feature maps from different layers of the neck and the head network are merged by concatenation to combine the multi-scale information. ELAN-GS is an improved part by replacing the ELAN normal convolution in YOLOv7 with GSConv. Detect is the last layer of the head network, which is used to generate the output of object detection. It accepts feature maps from different scales and uses anchors for object detection, generating detection boxes along with the corresponding category confidence and position information for each box.

### 3.2 BiFormer attention mechanism in the Backbone

In orchards, the texture features of citrus are characterized by low resolution, limited pixel area, tiny objects and so on. In addition, the texture features of XinHui citrus show a dense distribution. In this paper, the feature fusion part is enhanced by adding the BiFormer attention mechanism, which adaptively adjusts attention weights based on the features of the input image, allocating different levels of attention to different positions or features. In the prediction stage of the YOLOv7 model, the anchor boxes frames generated by prediction rely on NMS (non-maximum suppression) to filter out a large number of low confidence boundaries, which often results in misjudgment of citrus varieties due to low-resolution images with poor pixel area. The design of the BiFormer attention aims to decrease the model’s reliance on external information and utilize the original feature information to encode as much correlated information as possible for different locations, achieving attention focus.

**Figure 4:**
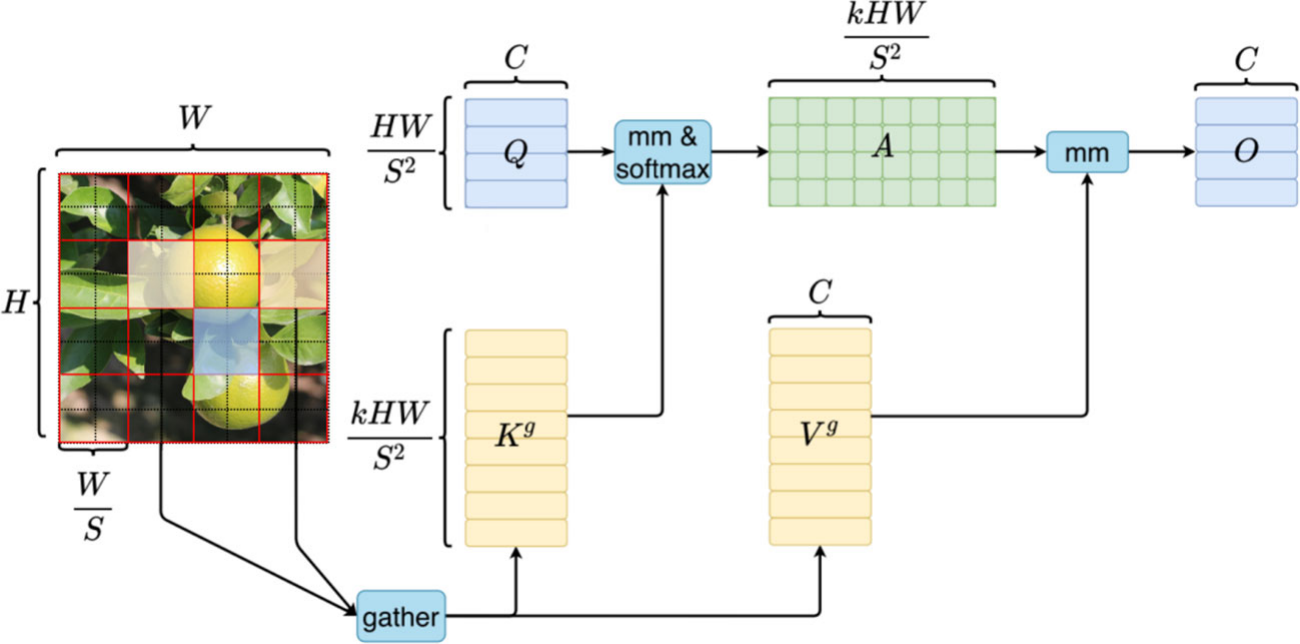
Computational schematic of BiFormer.

BiFormer is a variant of the Transformer model BiFormer introduces a dynamic attention mechanism into the original Transformer Model to enable more flexible content-aware computational allocation through bi-layered routing, and to allow the model to possess a sparsity of dynamical query-awareness. To this end, we proposed to add the BiFormer attention mechanism to YOLOv7 to strengthen the model’s ability to focus on textural features, as shown in **Figure 4**. It adds information of trivial objects by associating their perceptual features with the scene, and uses broader contextual information in the scene to assist in inferring the location or class of the trivial objects. Specifically, the core of the BiFormer attention module is BRA (Bi-level Routing Attention), which consists of region partition and input projection, region-to-region Routing with Directed Graph, and region-to-regional Routing with Directed Graph and Token-to-token Attention.

The concrete representation of attention: Queries Q∈ ${\text{R}}^{{\text{N}}_{\text{q}}\times \text{C}}$ , Key K∈ ${\text{R}}^{{\text{N}}_{\text{m}}\times \text{C}}$ , value vector Value V∈ ${\text{R}}^{{\text{N}}_{\text{m}}\times \text{C}}$ as input, R denotes the domain of real numbers as in Equation (1)


(1) $$\begin{array}{c} Attention\left(Q,K,V\right)=softmax\left(\frac{QK^{T}}{\sqrt{N}}\right)V \end{array}$$

H (height), W (width) and C (channel) denote the height, width and number of channels of the input image, respectively. Q, K, and V are numeric vectors, and the Softmax function maps the input to (0, 1) space, and $\sqrt{\text{N}}$ is a scalar.

We will introduce three parts of BRA, the first part is the “Region partition and input projection”. The input feature mapping is first divided into S × S disjoint regions, and then the query, key, and value vectors are obtained by dividing the linear projection of X, as in Equation (2):


(2) $$\begin{array}{c} Q=X^{r}W^{q},K=X^{r}W^{k},V=X^{r}W^{v} \end{array}$$

Q, K, V, $X^{r}$ ∈ $R^{{\text{S}}^{2}\times \frac{\text{HW}}{{\text{S}}^{2}}\;\times \text{C}}$ ; $W^{q},W^{k}$ and $W^{v}$ ∈ $R^{C\times C}$ are the weights of each linear projection.

The second part of BRA, “Region-to-region routing with directed graph”, is presented next. This part computes the regions that should be focused on by constructing a weighted directed graph from the depicted regions of the input feature graph X. First, the mean values of Q and K in each partition are computed separately to obtain $\;Q^{r}$ and $k^{r}$ ∈ $R^{S^{2}\times C}$ . Then, the adjacency matrix $A^{r}$ for the semantic correlations between the regions is computed using Equation (3):


(3) $$\begin{array}{c} A^{r}=Q^{r}{\left(k^{r}\right)}^{T} \end{array}$$

In order to reduce the number of interactions each region has with other regions, BRA keeps for each region by indexing matrix ∈o retain the K most relevant query regions as shown in Equation (4):


(4) $$\begin{array}{c} I^{r}=topkIndex\left(A^{r}\right) \end{array}$$

In the third part of the BRA, “Token-to-token attention”, key and query are integrated for GPU (Graphics Processing Unit) operations as shown in Equation (5):


(5) $$\begin{array}{c} K^{g}=g\left(K,I^{r}\right),V^{g}=g\left(V,I^{r}\right) \end{array}$$

where g(·) is the operation of collecting the tensor.

Therefore, we can represent the BRA according to the Transformer self-attention defined by Equation (6):


(6) $$\begin{array}{c} BRA=Attention\left(\text{Q},K^{g},V^{g}\right)+LCE\left(\text{V}\right) \end{array}$$

Among them, LCE is a local enhancement operation of multi-scale token aggregation by deep convolutional networks. By adding the BiFormer dynamic attention mechanism, the background interference is reduced. Under the premise of maintaining efficient detection, more features are captured and the detection accuracy is improved. As a core building block of Vision Transformers, the attention mechanism is a powerful tool for capturing long-distance dependencies. BiFormer attention is a major attention module that combines both global and local attention mechanisms, which it utilizes simultaneously. Global attention allows the model to interact with information over the entire input feature map, while local attention allows the model to focus on specific local regions. This combination helps the model to capture both global and local feature information, thus improving the model’s performance.

### 3.3 GSConv in the neck

**Figure 5:**
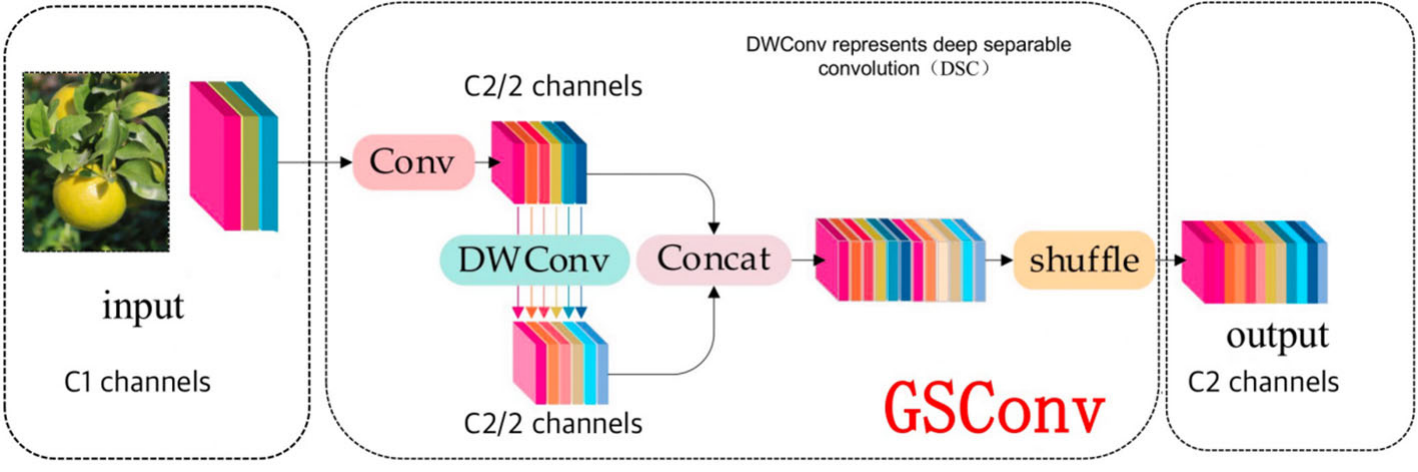
Structure of the GSConv module.

During object detection, more and more lightweight networks are being proposed in order to enable the deployment of algorithms into mobile scenarios. In the case of automatic picking devices in citrus orchards, lightweight neural network models are needed. Therefore, the neck of the YOLOv7 network model is improved to be lightweight by replacing the original convolution with GSConv in the ELAN of the neck. The GSConv module is a mixture of three convolutions: the standard convolution, the depth-separable convolution, and the Channel Shuffle as shown in **Figure 5**. The feature information generated by the standard convolution is infiltrated into each module of the feature information generated by the depth-separable convolution through the channel shuffle mixing strategy, so that the convolution calculation of the method is close to the output of the standard convolution. The non-linear representation of the feature information is enhanced by the addition of a depth-separable convolutional layer and a Shuffle layer, making the GSConv convolution more suitable for lightweight model detectors.

As the spatial information of the image is gradually converted into channel information when performing feature extraction on the image using convolutional neural network. Each spatial compression and channel expansion results in the loss of semantic information. Within channels, dense convolutional computation maximally preserves the hidden connections among each channel, but the sparse convolution between channels completely disrupts these connections. However, GSConv enables the output of convolution to be close to that of the original network, thus improving inference speed and accuracy with fewer parameters. GSConv is a special kind of convolutional operation in Convolutional Neural Networks (CNNs), which introduces the Channel Shuffle operation, aiming at fully mixing the information from the SC (Standard Convolution) into the DSC (Depth Separable Convolution) output to maximize the preservation of inter-channel connectivity information. Channel Shuffle is a homogeneous mixing strategy that propagates the information generated by SC (standard convolution) to the output of DSC (deep separable convolution) by exchanging local feature information on different channels.

The input feature maps are first subjected to a SC (Standard Convolution) operation, in which the connection information between channels is preserved. Then, the information generated by SC is evenly mixed into the output of DSC (Deep Separable Convolution) by Channel Shuffle operation. This process helps to overcome the drawbacks of DSC by introducing more SC information into DSC. The final output feature map contains a mixture of information from SC and DSC, which improves the accuracy.

The GSConv module mainly consists of Conv module, DWConv module, Concat module and Shuffle module, and its mathematical expression is (Equation 7), $f_{shuffle}$ denotes the shuffle operation, $f_{conv}$ consists of a standard convolution, a batch normalization operation and an activation function consists of a standard convolution, a batch normalization operation and an activation function, and $f_{dsc}$ denotes a depth-separable convolution (DSC), a batch normalization operation and an activation function.


(7) $$\begin{array}{c} X_{out}=f_{shuffle}\left(cat\left(f_{\text{conv}}\left(X_{in}\right),f_{dsc}\left(f_{conv}\left(X_{in}\right)\right)\right)\right) \end{array}$$

We embedded the GSConv module into the feature fusion stage, allowing us to reduce the number of parameters while maintaining high accuracy in our model. We did not use GSConv in the neck network because it would lead to deeper layers of the neck network, and a deeper network would exacerbate the resistance to spatial information flow (Hou et al., 2022).

## 4 Experimental results and discussion

The training and testing of this research work were experimented using a computer having an Ubuntu22.04LTS operating system, Core i9–9900 CPU @ 64-bit 4.90 GHz, 24 GB RAM (NVIDIA GeForce RTX 3090 GPU), python 3.8.18 and torch-1.11.0+cu113. The YOLOv7-BiGS including other compared models used in this paper received an input image of 640 × 640 pixels, 1 batch size, 0.937 momentum, 0.0005 weight decay, 0.2 IoU, 0.015 hue, 0.7 saturation, 0.4 lightness, 1.0 mosaic, 0.9 scale, 0.2 translate, 0.15 mix-up, and 150 epochs for training. Random initialization technique was utilized to initialize the weights for training all the models from scratch.

### 4.1 Evaluation of neural network model metrics

The relevant indicators for evaluating the effectiveness of neural network models are as follows (Sirisha et al., 2023): Precision, Recall, and AP. For binary classification problems, samples can be divided into four types: true positive (TP), false positive (FP), true negative (TN), and false negative (FN). The Equations (8) and (9) for Precision (P) and Recall (R) are as follows:


(8) $$\begin{array}{c} P=\frac{TP}{TP+FP} \end{array}$$

(9) R=TP/(TP+FN)

Average Precision (AP) is the average precision of the model, and AP is the area under the precision recall curve. Mean Average Precision (mAP) is the average value of AP. K is the number of categories. The Equations (10) and (11) for AP and mAP are as follows:


(10) $$\begin{array}{c} AP=\int_{0}^{1}\text{ρ}\left(\text{γ}\right)\text{dγ}\; \end{array}$$

(11) $$\begin{array}{c} mAP=\frac{1}{\text{k}}\sum_{\text{i}=1}^{\text{k}}{\text{AP}}_{\text{i}} \end{array}$$

The evaluation metric used in the validation is mAP (0.50:0.95) to select the optimal model. We present the results of mAP (0.50:0.95) and mAP (0.50) on the test set. MAP (0.50:0.95) represents the average mAP on different IoU thresholds (from 0.5 to 0.95, in steps of 0.05), and mAP (0.50) represents the average mAP on 0.5.

Params is used to measure the model complexity. Layer is a network topology of the model. GFLOPs is the speed of the model based on computation costs. Size measures the model weight. K is the convolution is kernel size, o is the output size, and H × W is the size of the outputted feature map. The Equations (12) and (13) for Params and GFLOPs are as follows:


(12) $$\begin{array}{c} Params=\left[i\times \left(k\times k\right)\times o\right]+o \end{array}$$

(13) $$\begin{array}{c} GFLOPs=H\times W\times Params \end{array}$$

### 4.2 Experimental results

**Figure 6:**
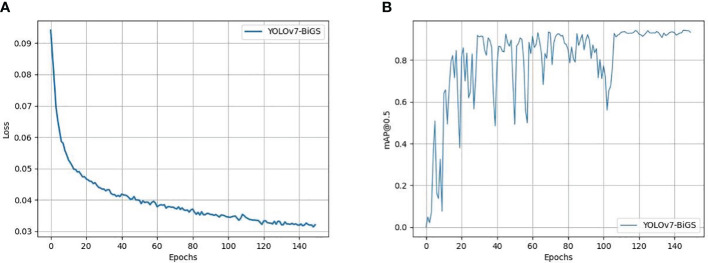
The Loss map and training outcome of models. **(A)** Loss map for model training and validation. **(B)** The training outcome of models.

Deep learning models are often referred to as black boxes due to their intricate architecture and multitude of parameters, rendering their internal mechanisms obscure. This lack of transparency presents significant hurdles in both training and assessing these models. To tackle this challenge and ensure the credibility of training and evaluation procedures, this paper undertook a comprehensive analysis of the loss function. **Figure 6A** depicts this paper meticulous monitoring of the loss function values throughout the training phase, with dedicated plots for both the training and validation datasets. Remarkably, the trends delineated in **Figure 6** signify a consistent convergence of the model as the training iterations progress. As the model undergoes learning, its performance steadily improves. The declining validation loss depicted in **Figure 6** correlates with an increasing mAP, as illustrated in **Figure 6B**. This convergence serves as compelling evidence bolstering the validity of our model and affirming the efficacy of our training and evaluation methodologies.

**Figure 7:**
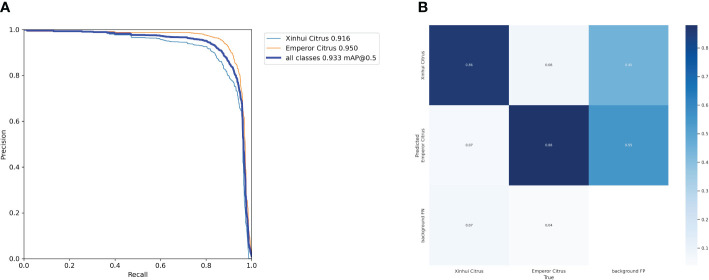
The Precision–Recall curve and confusion matrix of models. **(A)** Precision–Recal curve **(B)** Confusion matrix.

Based on the experimental results, this paper calculated the precision and recall at different thresholds and connected the points to form a PR curve, as shown in **Figure 7A**. The closer the curve is to the top-right corner, the less noticeable the decrease in precision as recall increases, indicating better overall performance of the model. **Figure 7B** presents the confusion matrix summarizing the prediction results for the classification. It can be observed that the true positive rates for Xinhui Citrus and Emperor Citrus are 86% and 88%, respectively. The proportion of false positives is very small, being 8% and 7%, respectively. Occasional instances of false negatives may be attributed to a high proportion of occlusions and the influence of complex environmental factors, which can impact the performance of the model. Overall, the classification of citrus varieties is accurate and comprehensive.

#### 4.2.1 Comparison experiments

Comparison experiments were conducted to evaluate the performance of classical object detection algorithms from the YOLO family, including Citrus-YOLOv7 (JChen et al., 2022) and the YOLOv7-BiGS algorithm. Citrus-YOLOv7 is enhanced with the CBAM attention mechanism and GhostConv model. YOLOv7-BiGS is improved by GSConv and BiFormer, and the results are obtained from the augmented test dataset, which are shown in **Table 1**. In the test set, after data augmentation, the number of images increased from 40 to 297. In this dataset, two varieties of citrus, Emperor Citrus and XinHui citrus, are included, and the ability of varietal classification is also tested. **Table 1** shows the experimental results of the improved model and different YOLO models on the self-constructed citrus dataset.

**Table 1:** Detection results of YOLOv7-BiGS, YOLOv5, YOLOv7, Citrus-YOLOv7 and YOLOv8 in the test set after data enhancement.

Model	Precision(%)	Recall (%)	mAP@.5 (%)	mAP@.5:.95(%)
YOLOv5	0.896	0.821	0.921	0.582
YOLOv7	0.852	0.821	0.889	0.591
YOLOv8	0.887	0.838	0.924	0.609
Citrus-YOLOv7	0.87	0.855	0.911	0.564
YOLOv7-BiGS (this paper)	0.91	0.873	0.937	0.619

Analysis of the experimental results pertaining to the five object detection models - YOLOv5, YOLOv7, YOLOv8, Citrus-YOLOv7 and YOLOv7-BiGS - as presented in **Table 1**, delineates the following observations: YOLOv7-BiGS exhibits superior performance across precision, recall, and mAP@0.5 metrics, values of 0.91, 0.873, 0.937, and 0.619, respectively. Citrus-YOLOv7 demonstrates precision, recall, and mAP@0.5 metrics, with values of 0.87, 0.855, 0.911, and 0.564, respectively. Comparative analysis reveals the exceptional accuracy and detection capabilities in YOLOv7-BiGS when contrasted with other models. Integration of the BiFormer attention mechanism and GSConv into the YOLOv7 network structure yields marked enhancements in YOLOv7-BiGS: a 5.8% elevation in Precision, a 4.8% increase in mAP, and a 5.2% improvement in Recall, indicative of reduced misclassification of background elements as citrus. Empirical evidence affirms that the refined YOLOv7-BiGS exhibits heightened resistance to interference, enabling more robust detection of citrus-specific features. Thus, the integration of the BiFormer attention mechanism and GSConv into the YOLOv7 network structure stands validated as an effective improvement strategy.

**Figure 8:**
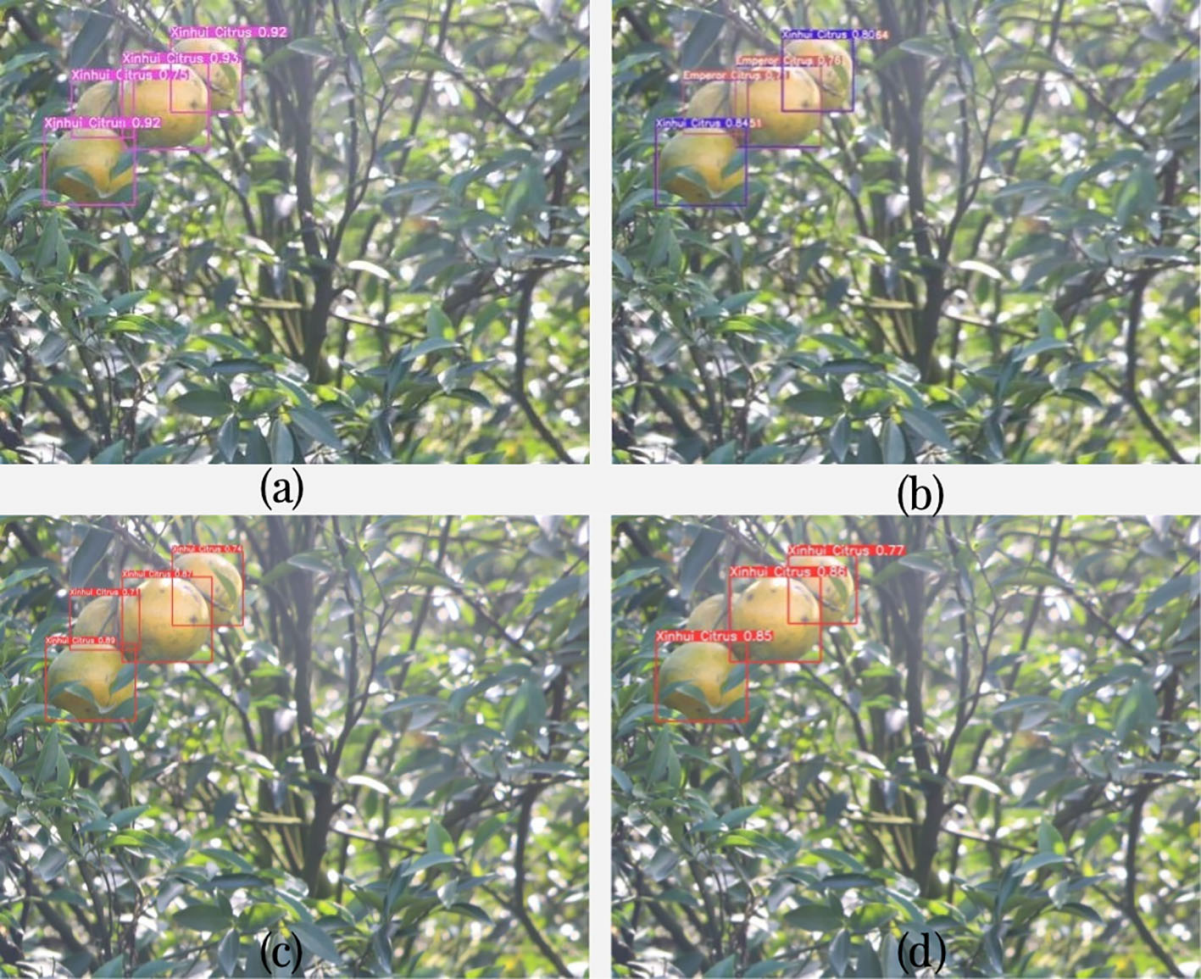
Comparison of object detection for the same image under occlusion. **(A)** shows the detection results of YOLOv7-BiGS; **(B)** shows the detection results of YOLOv7; **(C)** shows the detection results of YOLOv5; **(D)** shows the detection results of YOLOv8.

Some of the detection results are shown in **Figures 8A–D**. **Figure 8A** shows the detection results of YOLOv7-BiGS, **Figure 8B** shows the detection results of YOLOv7, **Figure 8C** shows the detection results of YOLOv5, and **Figure 8D** shows the detection results of YOLOv8. In the same picture, there are small objects with inconspicuous features at the same time, as shown in **Figure 8**, due to the poor quality of the pictures taken, the differences between the two kinds of citrus are small and difficult to be recognized easily, and clearer features are needed to be recognized, which leads to the misjudgment of the results of YOLOv7 variety detection. However, the confidence level of the improved YOLOv7-BiGS detection results is also very impressive, and is capable of extracting features with a high confidence level, moreover, it can also recognize the citrus that is obscured due to overlapping fruits. YOLOv7-BiGS was able to accurately recognize the variety category while overcoming poor image quality. However, in practice, the unpredictable light conditions are difficult to standardize, which these conditions affect variety detection. Therefore, the poor quality of the pictures meets the practical application scenarios and better highlights the generalization ability of the YOLOv7-BiGS method.

**Figure 9:**
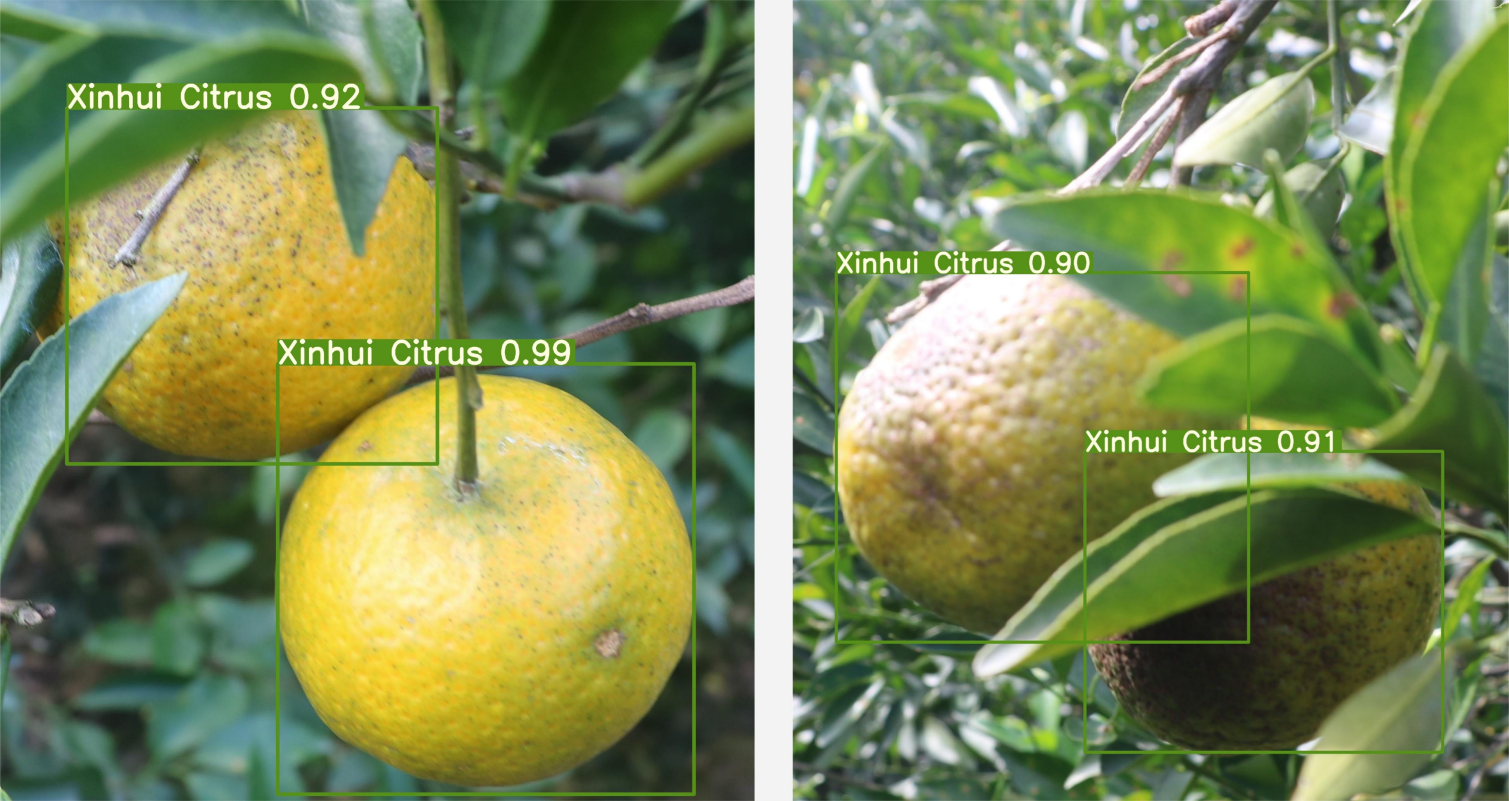
Results of YOLOv7-BiGS for the detection of XinHui citrus.

**Figure 10:**
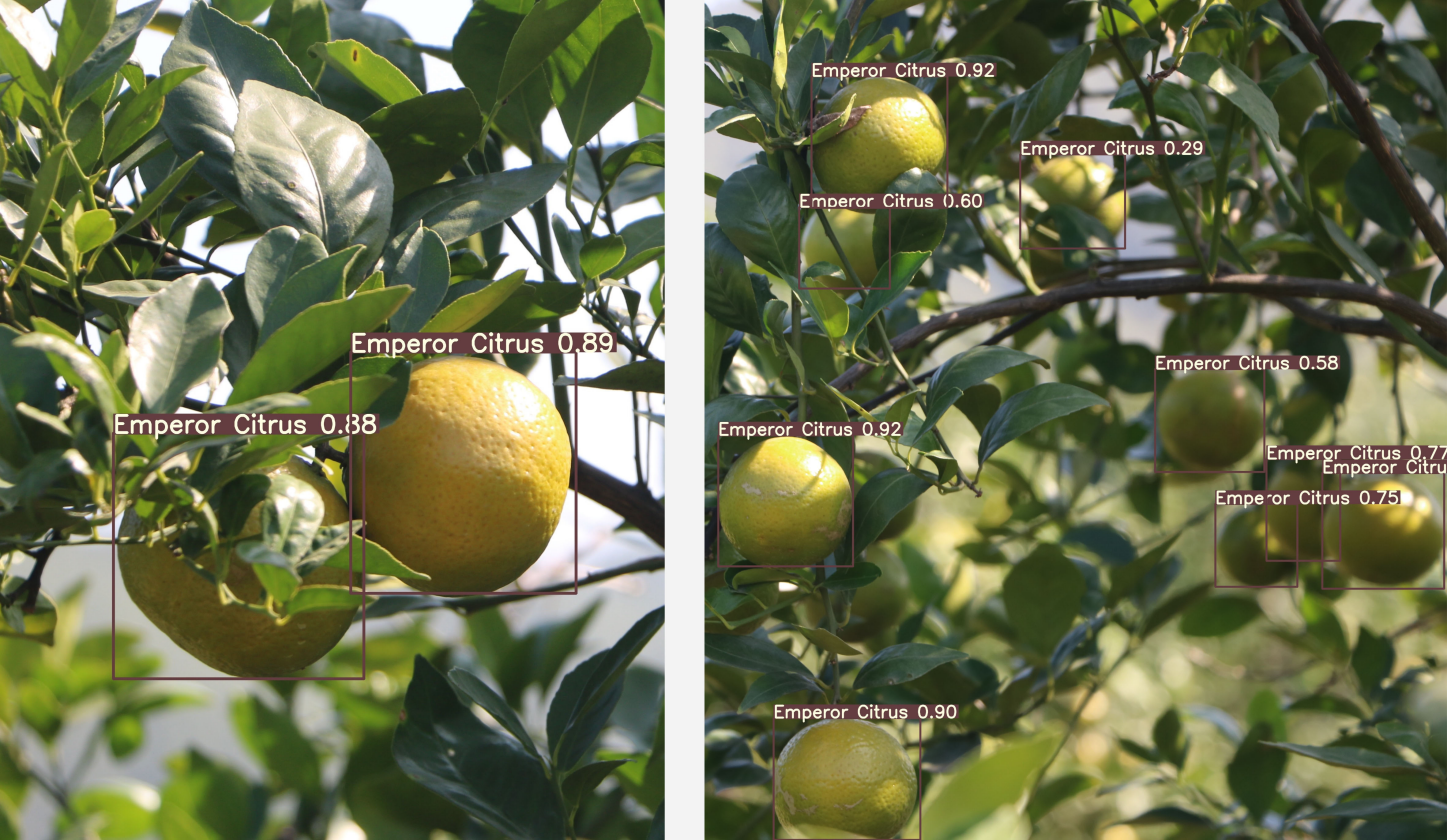
Results of YOLOv7-BiGS for the detection of Emperor citrus.

The dataset used in the results of **Table 1** already includes the XinHui citrus and Emperor citrus. Accuracy indicates the identification rates of various models for citrus varieties. The results of YOLOv7-BiGS, as shown in **Figures 9** and **10**, demonstrate its classification results for XinHui citrus and Emperor citrus in real natural environments. The study indicates that YOLOv7-BiGS can detect citrus varieties with high confidence and successfully complete the classification task. Therefore, considering the slight differences in features and details among different citrus varieties, the YOLOv7-BiGS citrus variety recognition model can extract richer citrus features, thereby accomplishing the task of variety detection.

#### 4.2.2 Ablation experiment

The initial experimentation on the citrus dataset utilized YOLOv7 as the baseline model. The findings indicated that YOLOv7 performed well in detecting clear, medium-sized, and large targets. However, there remained room for improvement in detecting partially obscured and unclear targets. Consequently, an attention mechanism was introduced into YOLOv7 to enhance the model’s feature extraction capabilities.

This paper adopts an approach combining ablation experiments and comparative experiments to validate the effectiveness of the proposed algorithm. Ablation experiments, as depicted in **Table 2**, are conducted to dissect and verify the efficacy of the improvements made. Through the ablation experiment, components were added sequentially and the improved network performance after adding components was compared to verify the necessity of the corresponding improvements. By training the data set after data enhancement, the weights generated after training are utilized to test the test set. First of all, the standard convolution in ELAN is replaced with GSConv based on YOLOv7. Meanwhile, the original standard convolution is replaced with Partial Conv (Chen et al., 2023) in the YOLOv7 network module ELAN, and the performances of the networks after the replacement of convolution are compared respectively. Finally, the BiFormer attention mechanism is added to the high-performance convolutional model to compare the performance. The results of the comparison with the original algorithm YOLOv7 are shown in **Table 2**.

**Table 2:** Results of ablation experiments of YOLOv7-BiGS on the test set after data enhancement.

Model	Precision	Recall(%)	mAP@.5(%)	Parameters
YOLOv7	0.852	0.821	0.889	36487166
YOLOv7+Partial Conv	0.878	0.859	0.854	31978494
YOLOv7+GSConv	0.885	0.881	0.934	34211966
YOLOv7+BiFormer+GSConv	0.91	0.873	0.937	36679038

Based on the experimental results in **Table 2**, when Partial Conv and GSConv were separately integrated into YOLOv7, the precision achieved was 0.878 and 0.885, respectively, accompanied by a reduction in parameters for both adaptations. Upon comparison, the addition of GSConv exhibited higher precision than the inclusion of Partial Conv, surpassing the original model’s metrics. Therefore, the performance of the network model improved notably after the incorporation of GSConv. Post the GSConv integration, to enhance the neural network’s ability to extract citrus features, the BiFormer attention mechanism was embedded into the ELAN layer of the Backbone, resulting in the creation of the YOLOv7-BiGS neural network model. Experimental results indicate that despite a slight increase in computational load, YOLOv7-BiGS achieved the highest precision.

#### 4.2.3 Object detection capability of YOLOv7-BiGS under partial occlusion

**Figure 11:**
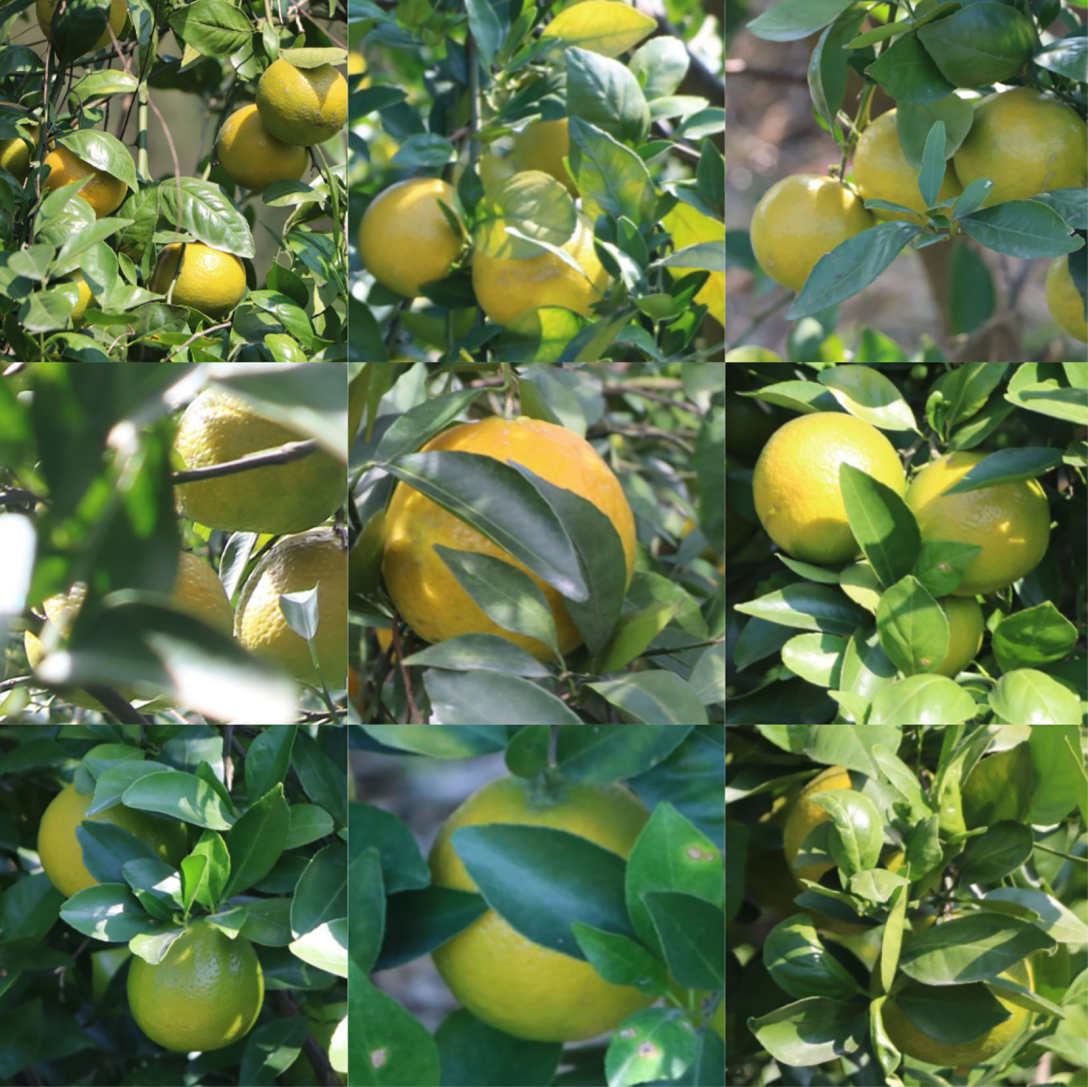
The partially occluded citrus test set.

In order to verify the real object detection ability of the improved model YOLOv7-BiGS in natural environments, the test results of YOLO series models are compared under occlusion respectively. A total of 26 occluded images are used as the test set. The partially occluded citrus test set is shown in **Figure 11** and the test results are shown in **Table 3**.

**Table 3:** Experimental results of different models in partial occlusion.

Model	Precision
YOLOv7	0.977
YOLOv5	0.873
YOLOv8	0.86
YOLOv7-BiGS	0.991

**Figure 12:**
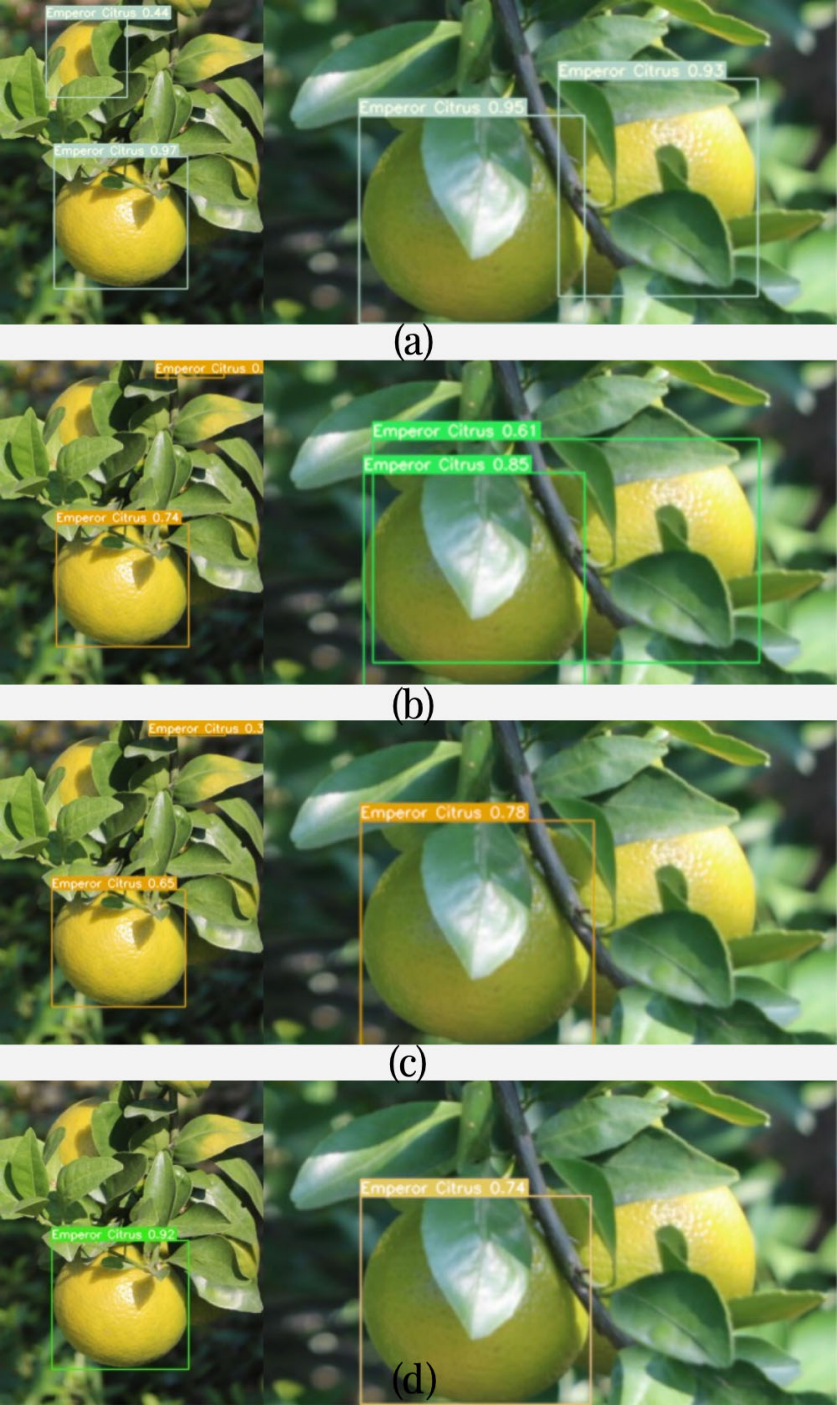
The detection results of different object detection models on the same image. **(A)** shows the detection results of YOLOv7-BiGS; **(B)** shows the detection results of YOLOv7; **(C)** shows the detection results of YOLOv5; **(D)** shows the detection results of YOLOv8.

From **Table 3**, it can be observed that YOLOv7-BiGS has the highest accuracy in detecting partially occluded citrus fruits, reaching 0.991. Two of the citrus images is selected from the test set, as shown in **Figure 12**. The comparison of object detection for the same image under occlusion is shown in **Figure 12A** for YOLOv7-BiGS, **Figure 12B** for YOLOv7, **Figure 12C** for YOLOv5, and **Figure 12D** for YOLOv8. From the detection results in **Figure 12**, it can be seen that the improved YOLOv7-BiGS algorithm can effectively detect the citrus object detection. Meanwhile, it can also recognize the citrus that is not fully exposed due to the leaf cover. In **Figures 12B–D**, these algorithms fail to detect the citrus that is more than 50% obscured by the leaves. As in **Figure 12A**, YOLOv7-BiGS was able to recognize citrus with overlapping fruits. In **Figures 12B–D**, these algorithms failed to detect the overlapped citrus or produced false positives. From the comparative detection results, it can be seen that YOLOv7-BiGS has the highest precision of 0.991 under occluded environments. Therefore, the improved algorithm effectively increases the detection rate of citrus objects in branch and leaf occlusion and object overlapping citrus images.

## 5 Conclusions

The paper proposes a non-destructive identification method for citrus varieties, which can automatically classify citrus fruits with similar appearances to improve the accuracy of variety detection. Initially, the authors collected images of various citrus varieties and constructed a dataset consisting of 3060 images by altering brightness, contrast, and adding noise. Secondly, they utilized YOLOv7 as the base network, integrating the BiFormer attention mechanism into the ELAN in the Backbone, and replacing the original convolution with GSConv in the Head. The main conclusions are as follows:

(1) YOLOv7-BiGS successfully accomplishes the variety detection task, and outperforms YOLOv7 in citrus variety detection performance. YOLOv7-BiGS achieves a mean Average Precision (mAP) of 93.7%, which is a 4.8% improvement over the original YOLOv7 model.
(2) Through ablation experiments, it is demonstrated that the combination of GSConv and BiFormer with YOLOv7 achieves the best performance.
(3) Compared with YOLOv5, YOLOv7, and YOLOv8, YOLOv7-BiGS exhibits better detection capability in complex environments.

The research findings indicate that the YOLOv7-BiGS model performs well in citrus variety detection tasks, providing technical support for smart agriculture, particularly in the breeding of citrus varieties like Chenpi. Additionally, variety identification technology can offer more efficient methods for automated management techniques such as automated fertilization in orchard management, promoting intelligent management of orchards.

## Data availability statement

The original contributions presented in the study are included in the article/supplementary material. Further inquiries can be directed to the corresponding author.

## Author contributions

FD: Funding acquisition, Supervision, Writing – original draft. JC: Data curation, Formal analysis, Methodology, Resources, Software, Visualization, Writing – original draft. LF: Funding acquisition, Methodology, Supervision, Validation, Writing – review & editing. JZ: Data curation, Formal analysis, Supervision, Writing – original draft. WQ: Data curation, Formal analysis, Writing – review & editing. JLL: Data curation, Formal analysis, Supervision, Writing – review & editing. JWL: Data curation, Formal analysis, Writing – review & editing. NL: Data curation, Formal analysis, Writing – review & editing.
